# Innovative Use of Olive, Winery and Cheese Waste By-Products as Novel Ingredients in Weaned Pigs Nutrition

**DOI:** 10.3390/vetsci10060397

**Published:** 2023-06-16

**Authors:** Georgios Magklaras, Ioannis Skoufos, Eleftherios Bonos, Anastasios Tsinas, Christos Zacharis, Ioannis Giavasis, Kostas Petrotos, Konstantina Fotou, Konstantina Nikolaou, Konstantina Vasilopoulou, Ιlias Giannenas, Athina Tzora

**Affiliations:** 1 Laboratory of Animal Science, Nutrition and Biotechnology, Department of Agriculture, School of Agriculture, University of Ioannina, Kostakioi Artas, 47100 Arta, Greece; 2 Laboratory of Animal Health, Food Hygiene and Quality, Department of Agriculture, School of Agriculture, University of Ioannina, Kostakioi Artas, 47100 Arta, Greece; 3 Department of Food Science and Nutrition, School of Agricultural Sciences, Karditsa Campus, University of Thessaly, 43100 Karditsa, Greece; 4 Department of Agrotechnology, School of Agricultural Sciences, Geopolis Campus, University of Thessaly, 41500 Larisa, Greece; 5 Laboratory of Nutrition, School of Veterinary Medicine, Faculty of Health Sciences, Aristotle University of Thessaloniki, 54124 Thessaloniki, Greece

**Keywords:** weaned pigs, novel silage, antioxidant status, microbial populations, intestine, meat quality

## Abstract

**Simple Summary:**

New sources of feeds are of high priority in modern animal production systems. In the present study, a novel silage created from Greek olive, winery, and feta cheese waste by-products, was evaluated as a feed ingredient at different inclusion rates (0%, 5% or 10%) for weaned pigs. The effects of this supplementation on the performance, health and meat quality parameters of the pigs were evaluated. The results showed no negative effects on the performance of the pigs. Some intestinal microbial populations were affected. The oxidative stability and the fatty acid profile of the meat was positively affected.

**Abstract:**

New sustainable sources of feeds, which can enhance the health and welfare of farm animals, lower feeding costs, and lead to safer products, are of high priority in modern animal production systems. In the present study, a novel silage created from Greek olive, winery, and feta cheese waste by-products, was evaluated as a feed ingredient at different inclusion rates (0%, 5% or 10%) in 34-day-old weaned pigs. The potential beneficial effects on performance, health and intestinal digesta microflora balance of the pigs were evaluated. Additionally, chemical, microbiological and quality analysis of the meat was carried out. Results showed no detrimental effects (*p* > 0.05) on the pigs’ performance and no significant changes (*p* > 0.05) in meat pH, color and chemical analysis. Ileum and cecum microflora populations (total anaerobes, Lactobacillaceae) were positively affected (*p* ≤ 0.05) by the dietary usage of the silage. The microbial populations (*Clostridium* spp.) of belly meat cuts were positively modified (*p* ≤ 0.01). The concentration of total phenols in the meat cuts were increased (*p* ≤ 0.05) and their resistance to oxidation was improved (*p* ≤ 0.05). In addition, the fatty acid profile of the meat lipids (polyunsaturated and n-3 fatty acids) was positively modified (*p* ≤ 0.001).

## 1. Introduction

In recent decades, farmers have been confronting multiple challenges especially those that applied intensive production systems. A major challenge is the constantly increasing cost of fodder and grains due global effects such as climate change, the reduction in natural feed sources and the extensive deforestation [1,2,3]. In addition, consumers today prefer healthy foods with beneficial nutritional properties that are produced in a sustainable way. Additionally, the goal for reduction in microbial resistance by minimizing use of antibiotics on farm animals has intensified the pursuit of novel feed ingredients and supplements of potential value [4]. Thus, researchers are seeking economical and innovative feeds for farm animals that may have increased added value by contributing to the production of functional foods of animal origin. Such novel feed ingredients could replace conventional feeds produced with excessive global environmental footprints and could improve the health and welfare of both the animals and the consumers.

Recently, unpredictable developments in the European feed trade market have attenuated the availability, and have vastly increased prices, of traditional protein and carbohydrate feed sources, such as soybean meal and cereals [5]. At the same time, in many countries, especially those of Southern Europe, large amounts of agro-industrial wastes are produced annually as by-products of various agro-industries such as olive oil production, cheese production and viticulture. These wastes are considered to be heavy environmental pollutants, not only because of their physicochemical status, but mostly because of the inappropriate way they are discarded by the industries into the ecosystem. Representative examples of these agro-industrial wastes in Greece are olive mill wastewaters, cheese whey (from feta cheese production) and grape pomace, all delivered in vast quantities [6,7,8]. However, it is well known that these agro-industrial wastes are not a risk for animals and humans if used as food or feed ingredients, as they have important nutritional value. They contain significant amounts of potentially useful bio-functional and nutritive components, including dietary fiber, unsaturated fatty acids, carotenoids, polyphenols, flavonoids, and useful biomass [9,10,11]. Since the methods and technologies of agro-industrial waste recycling are constantly evolving, incorporating such by-products in the diets of farm animals, should have high priority because it could provide substantial possibilities for the prospect of creating novel animal feeds with real benefits, for example the acceleration of sustainability models, the protection of natural animal health and welfare systems and the improvement of quality indexes of the animal products, especially concerning the antioxidant activity [12,13] and the lipid content [9,14,15].

Solid substrate fermentation, ensiling, and high solid or slurry processes are among the technologies available for protein enrichment of these wastes. Technologies for reprocessing these wastes must take into account the characteristics of individual wastes as well as the environment in which they are generated, reprocessed, and used [16,17]. It is typically impractical to include these wastes into feed formulas and production systems because of their physicochemical characteristics which impair feed digestibility and storage time. Even though silages are mainly incorporated in ruminant diets, novel silages made from waste by-products are also being tested in monogastric livestock, such as poultry and pigs, with encouraging results [13,18,19,20,21].

The present study tested a novel silage that has been created by a Greek scientific research group [9], facing the common challenge of using agro-industry wastes of locally produced products such as olive mill wastewater solids, grape pomace solids and deproteinized feta cheese way solids. This silage was created through testing for the optimal combinations of the three by-products [9] and has already been studied in broiler chickens [21]. The aims of this study were to examine if this silage could be used as a feed ingredient in weaned pigs’ diets and if it would benefit the pig production and health parameters, as well as the quality characteristics of the produced meat.

## 2. Materials and Methods

### 2.1. Experimental Design, Animals and Diets

The design and production of the innovative silage that was created from agro-industrial by-products and tested as a feed additive in this trial, was described in detail in the work published by Petrotos et al. [9]. In brief, multicriteria optimization mathematical models were used to evaluate the optimal mixing ratio of the three wastes: olive mill wastewater solids, grape pomace solids and deproteinized feta cheese way solids. After fermentation, an optimized silage was produced, with a low pH (pH = 4.37), a high lactic acid content (total acidity = 2.52 expressed as lactic acid), a high lactic acid bacteria count (total Lactic acid bacteria = 6.9 cfu/g) and a low yeast and mold count (total yeast and mold count = 0.1 cfu/g). Then, this silage was produced in large amounts, sufficient for the dietary trial with the pigs. Table 1 presents the chemical analysis of the tested silage.

All experimental procedures were in accordance with the National guidelines for animal trials (PD, 2013) and the authorities of the School of Agriculture of the University of Ioannina, Greece (UOI University Research Committee research registration: 61291). A veterinary surgeon and an animal scientist, both from the Department of Agriculture of the University of Ioannina, supervised the farming conditions and the weaned pigs during the whole experimental period. A total of 45 crossbreed weaned pigs (¼ Large White × ¼ Landrace × ½ Duroc) 34 days old were selected from a commercial pig farm in the Region of Epirus Greece and were randomly allocated to one of three treatment groups (Silage 0%, Silage 5% or Silage 10%). The initial pig body weights were similar in the three groups (average initial mean body weight 8.3 ± 0.12 kg) and each group contained the same number of females (7 per group) and males (8 per group). Each pig was individually marked with numbered plastic ear tags. The pigs in each treatment (N = 15) were housed in pens with slatted plastic floors, under controlled environmental conditions (ambient temperature, average humidity, ventilation rate, animal density) according to their production stage. All pigs were vaccinated against porcine circovirus type 2 (PCV2), *Mycoplasma hyopneumoniae*, *Actinobacillus pleuropneumoniae*, and Aujesky’s disease according to the standard management procedures of the farm. Ad libitum access to feed and water was available.

The control treatment (Silage—0%) was fed a commercial maize-based diet appropriate for weaned pigs, based on the recommended values from the National Research Council [22] and the database of Premier Nutrition [23]. The other two treatments were fed diets that incorporated either 5% (Silage—5%) or 10% (Silage—10%) of the tested novel silage, respectively. All three diets were formulated to be isonitrogenous and isocaloric. Table 2 presents the ingredients and chemical composition of the experimental diets.

The trial lasted 40 days (from 34 to 74 days of age) and during the experimental period, pigs were individually weighed on the mornings of the 1st, 22nd and 40th day, using a Mini–L 3510 scale for animals (Zigisis, Chalkidiki, Greece), while feed intake and mortality data were recorded daily. To estimate the effects of dietary treatments on the zootechnical performance indices of the pigs, the average gain (AG, kg per period), average feed intake (AFI, kg feed intake per period) and feed conversion ratio (FCR, kg feed intake/kg live weight gain) were calculated. On the last day of the trial blood samples were taken from six pigs per group and then these pigs were slaughtered under humanitarian conditions in a modern abattoir, close to the experimental farm.

### 2.2. Isolation, Enumeration and Identification of Bacterial Populations

Fresh digesta samples from the ileum and caecum were collected from six animals per treatment, immediately after slaughter. At first, 1 g of intestinal content was homogenized with 9 mL of sterile peptone water solution 0.1%. The Miles and Misra Plate Method (surface drop) was applied for the bacterial enumeration. Each sample was diluted serially via 12-fold dilutions (from 10-1 to 10-12) using standard 96-well plates for microdilutions. A total of 10 µL of each dilution was inoculated in media and incubated. MacConkey and Kanamycin aesculin azide (KAA) agar (Merck, Darmstadt, Germany), respectively, were used for the isolation of Enterobacteriaceae. All plates were incubated aerobically at 37 °C for 24–48 h. De Man, Rogosa and Sharpe (MRS) agar (Oxoid, Basingstoke, UK), M17 agar (Lab M Limited, Lancashire, UK) was used for the isolation and enumeration of Lactobacillaceae, while media were incubated at 37 °C for 48 h under anaerobic conditions. Bifidobacteriaceae isolation and enumeration were performed on transoligosaccharide propionate agar medium (TOS) (Merck, Darmstadt, Germany) supplemented with glacial acetic acid (1%, *v*/*v*) and mupirocin (100 μL/mL), and incubated anaerobically at 37 °C for 72 h. Total aerobic and anaerobic bacterial counts were determined using plate count agar medium (Oxoid, Basingstoke, UK), while plates were incubated at 30 °C aerobically for 48 h and at 37 °C anaerobically for 48–72 h, respectively. For bacterial counts, typical colonies from an appropriate dilution were counted on a microbial colony counter instrument and counts were expressed as colony forming units (CFU) × log per 1 g wet weight sample. Typical colonies grown on different media were then described and subcultured. All bacterial populations were identified at the family level by the automated Vitek 2 compact system (bioMérieux, Marcy l’Etoile, France), which provided reliable and accurate results for a large range of gram-positive and gram-negative bacteria [24]. For the identification of Enterobacteriaceae, Enterococcaceae, Lactobacillaceae and Bifidobacteriaceae, the VITEK 2 Gram-Negative identification card (ID-GN) (bioMérieux, Marcy l’Etoile, France), the VITEK 2 Gram-Positive identification card (ID-GP) (bioMérieux, Marcy l’Etoile, France), the CBC and ANC identification cards (bioMérieux, Marcy l’Etoile, France) and the VITEK 2 ANC ID card (bioMérieux, Marcy l’Etoile, France) were used, respectively.

### 2.3. Analysis of the Blood

For the determination of hematological and biochemical parameters, blood samples were collected from six pigs per treatment, prior to slaughter, on the last day of the trial (day 40). Feed was removed from the feeders 4 h before blood sampling. A sample of 4 mL of blood was collected from the jugular vein of the pigs and placed in vacutainer tubes with ethylenediamine tetra acetic acid (EDTA). Hematological parameters (hemoglobin; erythrocytes; hematocrit, HCT; leucocytes; lymphocytes) were determined using an automated analyzer MS4 (Melet Schloesing Lab, Osny, France) and biochemical parameters (albumin, ALB; alanine aminotransferase, ALT; aspartate aminotransferase, AST; cholesterol, CHOL; creatine kinase, CK; glucose, GLU; total bilirubin, TBIL; triglycerides; TRIG) in serum using the IDEXX VETTEST 8008 (IDEXX LAB, Westbrook, ME, USA).

### 2.4. Meat Quality

#### 2.4.1. Chemical Analysis, pH Measurement and Color Analysis of the Meat

Meat samples detached from the ham (biceps femoris muscles), shoulder (triceps branchii muscles) and belly (external abdominal muscles), were collected and immediately frozen at −20 °C for the meat chemical analysis. Each meat sample, weighing 200 g, was ground using an industrial large meat grinder (Bosch, Gerlingen, Germany). Moisture, crude protein, fat, collagen and ash content were determined by near infra-red spectroscopy using a FoodScanTM Lab (FOSS, Hilleroad, Denmark) in transmittance mode, according to AOAC 2007.04 for meat and meat products [25].

The pH measurement of shoulder, ham and belly samples was performed using a portable Hanna HI981036 instrument (Hanna Instruments, Woonsocket, RI, USA) pH meter for solid samples, measuring six pig meat samples from each group by inserting the stainless probe deep in the tissue. Average values from each group were estimated.

The color of the shoulder, ham and belly meat samples was evaluated according to the Hunter scale (L*, A*, B* values) by using the CAM–System 500 Chromatometer (Lovibond, Amesbury, UK).

#### 2.4.2. Oxidative Stability Analysis of the Meat

For the measurement of the total polyphenols of the pig meat samples, a modified Folin–Ciocalteu method was used. According to this method, 0.2 g/L of gallic acid (Merck, Germany) was diluted in 100 mL of distilled water. The stock solution was used to prepare the standards solutions 0.005, 0.01, 0.05, 0.1, 0.25, 0.5 and 1 g/L of gallic acid. From each standard solution 0.2 mL was transferred into a 50 mL falcon tube and mixed with 10.8 mL of distilled water, 8 mL of Na_2_CO_3_ (75 g Na_2_CO_3_ in 1 L distilled water) (Penta Chemicals, Prague, Czech Republic) and 1 mL of the Folin–Ciocalteu reagent (PanReac Applichem, Darmstadt, Germany). A control sample was prepared in which 0.2 mL of distilled water was added instead of a standard solution to calibrate the UV–Vis spectrophotometer (DR 5000, Hach Lange, Düsseldorf, Germany). All tubes were homogenized in a vortex mixer and were placed in a dark cabinet for 1 h at room temperature. After the incubation, the control was used to calibrate the UV–Vis spectrophotometer (DR 5000, Hach Lange) at 750 nm and then all the standards solutions were measured. A standard curve of concentration of gallic acid and absorbance was constructed using the Microsoft Excel software where the R2 was 0.9989. The above procedure was followed to measure the total polyphenols of the pig meat. Then, 5 g of shoulder, belly or ham meat were homogenized in a blender with 10 mL of distilled water and filtered with filter paper. A total of 0.2 mL of the filtrate was transferred in 50 mL falcon tubes and mixed with 10.8 mL distilled water, 8 mL of Na_2_CO_3_ (75 g/L solution) and 1 mL of the Folin–Ciocalteu reagent. A blank sample was prepared in which 0.2 mL was added instead of sample to calibrate the UV–Vis spectrophotometer. All tubes were mixed in vortex and placed in a dark cabinet at room temperature for 1 h. After the incubation, the blank sample was used to calibrate the spectrophotometer at 750 nm and then all the samples were measured.

For the measurement of lipid oxidation of pig meat samples, a modified version of the 2-thiobarbituric acid method (TBARS, thiobarbituric acid reactive substances) described by Dias et al. [26] was applied. In brief, 5 g of shoulder, ham or belly meat was homogenized with 25 mL of trichloroacetic acid in a blender and transferred to a glass bottle and left there for 20 min. Then, the samples were filtered with filter paper and 5 mL of the filtrate was transferred to glass tubes with 5 mL of 2-thiobarbituric acid. A blank sample was prepared by replacing the sample with 5 mL of trichloroacetic acid. All tubes were mixed in a vortex mixer and placed in water bath at 60 °C for 15 min. The samples were measured in a UV–Vis spectrophotometer after the calibration with the blank sample at 532 nm.

#### 2.4.3. Microbiological Analysis of the Meat

For the enumeration of the bacterial counts in meat, the average values from six individual pigs from each group were estimated for each microbial population of meat samples after a 2-day refrigerated storage at 4 °C after slaughter, which was necessary for transportation and handling of samples prior to microbiological analysis. More specifically, 10 g of shoulder, belly or ham meat were homogenized in Bagmixer 400 (Interscience, Saint-Nom-la-Bretèche, France) with 90 mL of sterile Maximum Recovery Diluent (MRD) (Oxoid, Basingstoke, UK). Each sample was 10-fold diluted using glass tubes with 9 mL of sterile MRD. Appropriate dilutions were inoculated, either 1 mL or 0.1 mL, in petri dishes for the enumeration of the bacterial count. The tested microorganisms were: *Escherichia coli*, which was cultivated on tryptone bile X-glucuronide (TBX) agar (Oxoid, Basingstoke, UK) and incubated aerobically at 37 °C for 24 h; sulfite reducing clostridia which were counted on perfringens agar base (Oxoid, Basingstoke, UK) incubated at 37 °C for 48 h under anaerobic conditions using anaerobic jars with the addition of Anaerocult A (Oxoid, Basingstoke, UK); *Staphylococcus aureus* and *Staphylococcus* spp. which were spread on Baird Parker agar (Oxoid, Basingstoke, UK) supplemented with egg yolk tellurite (50 mL/1 L substrate) and incubated under aerobic conditions at 37 °C for 48 h. Total Mesophilic count was measured in Plate Count Agar (PCA) (Oxoid, Basingstoke, UK) at 30 °C for 48 h under aerobic conditions and *Campylobacter jejuni* was spread on Campy Blood Free Selective Medium (CCDA) (Acumedia–Lab M, Lansing, MI, USA) with Campylobacter selective supplement under microaerophilic conditions in an incubator with 10% CO_2_ at 37 °C for 72 h.

All samples were examined for the presence of *Salmonella* spp. and *Listeria monocytogenes* per 25 g of shoulder, ham and belly meat using the ISO 6579:2002 and ΙSO 4833:2001 methods, respectively. The petri dishes were incubated in Binder BD 115 thermostable incubators.

#### 2.4.4. Fatty Acid Analysis of the Meat

For shoulder and belly meat fatty acid analysis, samples were processed as recommended by O’Fallon et al. [27]. Separation and quantification of the methyl esters was performed according to the method described by Skoufos et al. [28], with the use of a TraceGC (Model K07332, Thermofinigan, Thermoquest, Milan, Italy) equipped with a flame ionization detector.

### 2.5. Statistical Analysis

The basic study design was RCB (random complete block design), and each ear tagged pig was considered as the experimental unit. Microbiology data were log-transformed (Log_10_) prior to analysis. Data homogeneity was tested using Levene’s test. Experimental data were analyzed by one-way analysis of variance (one-way ANOVA) or the Krushar–Wallis Test, depending on the data format, using the SPSS v20 statistical package [29]. Tukey’s test was used for post-hoc comparisons between the three treatments. Significance level for all tests was set at 5% (*p* ≤ 0.05).

## 3. Results

### 3.1. Performance Parameters

The effects of the dietary use of the novel silage on pig performance parameters are presented in Table 3. Final body weight of the pigs did not differ (*p* > 0.05) between the three treatments. It was noted that the weight gain was higher for the Silage 10% treatment (*p* ≤ 0.05) compared to the Silage 5% treatment during the period of 22–40 days, however the weight gain for the overall period (1–40 days) did not differ significantly (*p* > 0.05) between the three treatments, despite that the pigs were in the crucial post-weaning period of their life. Feed intake and feed conversion ratio were within the expected ranges for the commercial pig farm that housed the experimental trial. Concerning the carcass parameters, carcass weight and dressing percentage did not differ (*p* > 0.05) between the three treatments.

### 3.2. Intestinal Microflora

Intestinal microflora populations were affected by the dietary use of the tested silage (Table 4). In the ileum digesta it was noted that total anaerobes were increased (*p* ≤ 0.05) in the Silage 10% treatment, compared to the Silage 5% treatment. Lactobacillaceae (*p* ≤ 0.001) were increased in the control Silage 0% and the Silage 10% treatments compared to the Silage 5% treatment. In the caecum digesta, total anaerobes were lower (*p* ≤ 0.001) in treatments Silage 5% and Silage 10% compared to the control treatment, while Lactobacillaceae were lowest (*p* ≤ 0.001) in the Silage 10% treatment, intermediate in the Silage 5% treatment and highest in the control the Silage 0% treatment. The other evaluated microbial species did not differ (*p* > 0.05) between the three treatments.

### 3.3. Blood Parameters

The effect of the novel silage on pig blood hematological and biochemical parameters is presented in Table 5. Concerning hematological values, the monocyte levels were significantly higher (*p* ≤ 0.01) in the Silage 10% treatment, compared to the other two treatments. Additionally, hematocrit (Hct) and hemoglobin (Hb) levels were lower (*p* ≤ 0.05) in the Silage 10% group, compared to the control Silage 0% treatment. The other hematological parameters did not differ (*p* > 0.05) between the treatments. Furthermore, regarding the biochemical parameters, blood glucose (GLU) was lower (*p* ≤ 0.05) in the Silage 10% treatment, compared to the control Silage 0% treatment. The other blood biochemical parameters did not differ between the three treatments.

### 3.4. Analysis of the Meat

As shown in Table 6, shoulder meat (triceps branchii) ash content was increased (*p* ≤ 0.05) in the Silage 10% treatment compared to the Silage 5% treatment, however the other chemical composition parameters (fat, moisture, protein and collagen) did not differ (*p* > 0.05). Regarding ham meat (biceps femoris), no differences were noted in any of examined parameters. In the belly meat (external abdominal) ash content was increased (*p* ≤ 0.01) in the Silage 5% and Silage 10% treatments, compared to the control Silage 0% treatment. The other chemical composition parameters (fat, moisture, protein and collagen) of the belly meat did not differ (*p* > 0.05). The pH values of all meat samples did not differ between the treatments (*p* > 0.05).

Regarding the color measurements (Table 7), there were no significant statistical differences (*p >* 0.05) in the shoulder and the ham meat samples.

Table 8 presents the results of the meat microbiological analysis. In the shoulder and the ham meat samples, no significant differences (*p >* 0.10) were identified between the three treatments. In the belly meat the sulfite reducing Clostridium (which include the pathogenic clostridia *C. perfringens* and *C. botulinum*) counts were lower (*p* < 0.05) in the Silage 10% treatment compared to the other two treatments, whereas the other examined microbial populations did not differ between the treatments. Finally, in all samples there was an absence of *Salmonella spp*. and *Listeria monocytogenes* (per 25 g of sample).

Data on total phenolic content and oxidative stability of meat samples are presented in Table 9. In the shoulder meat, the phenolic content was significantly higher (*p ≤* 0.05) in the Silage 10% treatment compared to the control Silage 0% treatment, whereas the TBARS analysis results did not differ between the treatments. In the ham meat, the phenolic content was significantly higher (*p ≤* 0.05) in the Silage 5% and Silage 10% treatments compared to the control Silage 0% treatment. Additionally, the TBARS content was significantly lower (*p ≤* 0.05) in the Silage 10% treatment compared to the control Silage 0% treatment. In the belly meat, the phenolic content was highest (*p ≤* 0.001) in the Silage 10% treatment, intermediate in the Silage 5% treatment and lowest in the control Silage 0% treatment whereas the TBARS level was significantly lower (*p ≤* 0.05) in the Silage 10% treatment.

Fatty acid analysis of the shoulder meat cuts is presented in Table 10. The percentages of most identified fatty acids differed (*p* < 0.05) between the control (Silage 0%) treatment and the two other supplemented treatments. Overall, the total saturated fatty acids (SFA) were lowest (*p ≤* 0.001) in the Silage 10% treatment, intermediate in the Silage 5% treatment and highest in the silage 0% treatment. The total monounsaturated fatty acids (MUFA) were highest (*p ≤* 0.001) in the Silage 5% treatment, intermediate in the Silage 0% treatment and lowest in the Silage 10% treatment. The total polyunsaturated fatty acids (PUFA) were higher (*p ≤* 0.001) in the supplemented Silage 5% and Silage 10% treatments compared to the control. The total omega-3 fatty acids were highest (*p ≤* 0.001) in the Silage 10% treatment, intermediate in the Silage 5% treatment and lowest in the Silage 0% treatment. The total omega-6 fatty acids were highest (*p ≤* 0.001) in the Silage 0% treatment, intermediate in the Silage 5% treatment and lowest in the Silage 10% treatment. Finally, the ratio of omega-6/omega-3 fatty acids was lowest (*p ≤* 0.001) in the Silage 5% treatment, intermediate in the Silage 10% treatment and highest in the Silage 0% treatment.

The results of the fatty acid analysis of the belly meat cuts are shown in Table 11. The dietary supplementation of the examined silage modified (*p* < 0.05) the fatty acid composition of most examined fatty acids, compared to the control Silage 0% treatment. Overall, the total SFA were lowest (*p ≤* 0.001) in the Silage 10% treatment, intermediate in the Silage 5% treatment and highest in the Silage 0% treatment. The total MUFA was lowest (*p ≤* 0.001) in the Silage 5% treatment, intermediate in the Silage 10% treatment and highest in the Silage 0% treatment. The total PUFA was highest (*p ≤* 0.001) in the Silage 5% treatment, intermediate in the Silage 10% treatment and lowest in the Silage 0% treatment. The total omega-3 fatty acids were highest (*p ≤* 0.001) in the Silage 10% treatment, intermediate in the Silage 5% treatment and lowest in the Silage 0% treatment. The total omega-6 fatty acids were highest (*p ≤* 0.001) in the Silage 5% treatment, intermediate in the Silage 10% treatment and lowest in the Silage 0% treatment. Finally, the ratio of omega-6/omega-3 fatty acids was lowest (*p ≤* 0.001) in the Silage 10% treatment, intermediate in the Silage 5% treatment and highest in the Silage 0% treatment.

## 4. Discussion

The ever-increasing demand for livestock products, due to urbanization, improvement of living standards, but also due to the rapid increase in the human population may create an increasing shortage of available feed resources by 2050 [30]. The effective and sustainable development of the livestock sector should include a reduction in wastage and furthermore a feasible reduction in production costs, by delivering an expansion of the feed resource base. Such goals can be achieved, at least in part, by finding new animal feeds, particularly those that do not compete with human foods.

In this trial, a novel silage made from olive, winery and cheese waste by-products was used as a feed ingredient for the diet of weaned pigs in a commercial farm in Greece to investigate its effects on their performance, health and meat quality characteristics. Cheese waste was incorporated in the initial design of the biofunctional silage mainly due to its high caloric and lactose content [31], grape pomace due to its abundance in phenolic compounds, unsaturated fatty acids, dietary fiber, and beneficial microorganisms [32], while olive mill waste water was included due to the presence of several compounds with demonstrated antioxidant and radical scavenging action, including hydroxytyrosol, oleuropein, tyrosol, caffeic acid, p-cumaric acid, verbascoside, and elenolic acid [33]. Similar agro-industrial by-products have been previously individually tested in several trials as ingredients of pig diets with encouraging results regarding the economical and biological effects: olive mill waste water solids [18,34,35], grape pomace solids [12,13,15,20] or cheese way solids [36,37,38,39]. To the best of our knowledge, it is the first time that such a combination of the previously mentioned three agro-industrial wastes, in a silage form, has been evaluated in weaned pig diets.

It has been reported that partially delactosed whey in the feed of non-ruminants can lead to increased body weight gain, enhanced feed efficiency and improved protein and fat digestibility [31]. On the other hand, Martins et al. [38] found no significant differences (*p* > 0.05) in the performance and carcass indices of growing pigs which were fed with cheese whey that substituted 0%, 20% or 30% of the dry substance of their rations. In trials using only grape pomace solids in pig diets, Hao et al. [12] and Wang et al. [20] reported no significant effects (*p* > 0.05) between control and grape pomace enriched dietary pig groups in their growth performance, average daily feed intake and feed conversion ratio. In contrast, Kafantaris et al. [15] noticed a significant increase (*p* < 0.05) in average daily gain of the piglets that consumed the grape pomace experimental diet, while Liehr et al. [35] demonstrated refined piglet growth due to improvements in intestinal integrity after consumption of olive-oil bioactive extracts. Our results, with the use of the bioactive silage, are partly in agreement with the above findings since it was noted that body weight gain was higher for the Silage 10% treatment (*p* ≤ 0.05) compared to the other two treatments during the period of 22–40 days, but with neither positive nor negative effects on the other performance and carcass parameters. This increased growth rate in the first half of the trial could potentially be attributed to the increased feed intake and better feed palatability, although further testing is necessary to confirm this.

One of the key variables influencing the content and operation of the gut microbiota of pigs is their diet [40]. The composition of the gut microbiome and, as a result, the products of bacterial metabolism have an impact on host health. Kafantaris et al. [15] reported a significant increase (*p* < 0.05) in lactic acid bacteria and a significant reduction (*p* < 0.05) in Enterobacteriaceae in piglets’ feces that were fed a grape pomace experimental diet for 30 days. The intestinal microbiota species of the groups that were fed grape pomace did not change when compared to the control group, but the ratio of beneficial bacteria increased [20]. In our study, intestinal microflora populations were affected by the dietary use of the novel silage fed at 5% or 10% concentrations. A significant (*p* < 0.05) reduction in total anaerobes in the ileum (Silage 5%) and caecum (both Silage 5% and Silage 10%) of the silage supplemented groups was recorded. In addition, the dietary silage supplementation modified the Lactobacillae counts in both the ileum and the caecum. Research on pigs fed diets containing grape pomace revealed that this supplementation can attenuate the number of Lactobacillaceae counts in the proximal colon [41] while other researchers [42] reported a positive impact of feeding apple pomace and red-grape pomace on Lactobacillaceae numbers and a tendency to attenuate total anaerobes [43]. In addition, the overall balance of other intestinal microbial families could potentially be modified such as Enterobacteriaceae, Enterococcaceae, and Bifidobacteriaceae, although such effects were not apparent in the present trial.

In the present study, hematological and biochemical parameters were analyzed as indications of general health status. Concerning hematological values, the monocyte levels were significantly higher in the Silage 10% treatment, while both hematocrit (Hct) and hemoglobin (Hb) levels were the lowest in this treatment. These results are in accordance with other studies where piglets were fed apple or red-grape pomace enriched diets and reported decreased hematocrit (Hct) and hemoglobin (Hb) levels and elevated monocytes [43]. Biochemical parameters were similar for all treatments except for blood glucose (GLU), which was lowest in the Silage 10% group. Similarly, Formigoni et al. [37] determined a significant reduction in plasma glucose and urea after feeding pigs with liquid whey.

The chemical composition of the meat in monogastric animals can be changed to a noticeable degree by dietary changes. In the present study, the dietary supplementation with the silage did not affect main meat chemical composition values, as fat, protein, collagen, moisture and pH did not differ significantly between the three treatments for all meat cuts. The only apparent difference was a small increase in the total ash content of the shoulder and belly, especially in the Silage 10% treatment. Moreover, the carcasses and meat cuts quality parameters were within acceptable limits for commercial use. The ash level of the meat is a significant component in determining its nutritional value, quality, and physicochemical characteristics while its content, along with protein, varies depending on water content [44]. Ash content has been positively correlated, and intramuscular fat negatively correlated, to increased lean meat percentage in pigs [45]. Our findings suggest that not only can the novel silage be utilized without affecting meat attributes, but it may also be a promising option for further research and use, to better understand the biological impacts of mineral deposition in muscle.

Color of meat is a major quality characteristic and a key factor for consumer preference. Marbling and color are used to assess the ‘value’ or quality of a meat cut. In the present study, there were no apparent differences in any of the examined color parameters (lightness, redness, yellowness). It has been reported that color can be affected by some feed ingredients, such as carotenoids and other pigments that can be found in plant material or the feed iron levels [46]. Moreover, meat color can be modified during storage through the combined effects of water loss, maturation and lipid oxidation. Previously, Tian et al. [47] indicated no significant effects on meat color parameters of pigs fed a 6% dried grape pomace powder, while other researchers noticed an increase in A* value which led to redness of pork meat [46] or even a rise in both A* and B* values (20% and 31%, respectively), for 21-day old piglets fed a 3% grape pomace solids inclusion rate [48].

Microbial growth in meat cuts is closely related to their quality and safety. In the present study, the identified microbial populations were low and within acceptable levels, and in all meat samples there was absence of *Salmonella* spp. and *Listeria monocytogenes* (per 25 g). The only apparent statistically significant effect was the reduction in sulphite-reducing clostridia (which include the pathogenic clostridia *C. perfringens* and *C. botulinum*) especially in the belly meat samples. The antimicrobial activity of grape pomace has been reported and is attributed to its flavonoid content and nonflavonoid (phenolic acids and stilbenes) compounds [49,50]. There is a link between gut microbiota, development and function of skeletal muscle and meat quality, implying that diet can influence microbial populations, bacterial metabolites, and meat quality [51,52]. The microbiota heredity has been estimated for carcass composition and meat quality traits in pigs, and positive microbial correlations have been found among different traits, particularly those related to meat color and firmness score [53]. It should be noted that in a previous trial that tested the same novel silage in broiler diets at the same inclusion levels (5% and 10%), the effect of this supplementation on broiler meat microbial populations was more noticeable [21]. This variability potentially highlights the biological and physiological differences in digestion, growth and tissue composition between different animal species, as well as the need to extensively test new products in different animal production systems.

Weaning is a critical event that can cause physiological, environmental, and social stress in pigs, increasing their risk of intestinal dysfunction and oxidative stress [54,55]. Lipid oxidation is closely related to the control of meat pathogenic or spoilage microflora, as well as to the quality and organoleptic properties of the meat products. In the present study, the total phenol content was elevated and the TBARS levels were reduced in the meat cuts of the supplemented treatments and these effects were more noticeable in the high silage inclusion (10%) treatment. It seems that there was a correlation between the dietary phenol content and meat resistance to oxidation. This is an important finding since lipid oxidation and rancidity directly affect meat quality and storability, especially during refrigeration or freezing of the meats. Polyphenols have the potential to perform as antioxidants, to scavenge free radicals, and to inhibit some enzymes involved in free radical production and thus stimulate an immune response [56]. Gerasopoulos et al. [18], fed a diet enriched with olive mill wastewater solids to piglets, and reported downregulated oxidative stress-induced lipid and protein damage, as demonstrated by a decrease in TBARS and CARB levels, respectively. Piglet diets containing 5% grape pomace were proven beneficial for the metabolism of normal blood constituents and overall health maintenance, as it increased polyphenol content in blood plasma and improved antioxidant activity in the liver, spleen, and kidneys [13]. Piglets fed an experimental diet containing 9% grape pomace solids showed less oxidative stress-induced damage to lipids and proteins, as confirmed by lower levels of TBARS and CARB in the grape pomace solids group compared to the control [15].

It has been well proven that the fatty acid composition of the lipids in the meat and other tissues of monogastric animals is directly affected by dietary lipids [21,57,58]. In the present trial, the meat fatty acid composition was modified to an extensive degree by silage supplementation. Overall, the supplemented treatments had elevated amounts of polyunsaturated fatty acids and especially the desirable omega-3 (n-3) fatty acids (docosapentaenoic acid, docosahexaenoic acid), which resulted in reduced omega-6/omega-3 (n-6/n-3) ratios. It has been documented that the inclusion of olive mill wastewater solids in ruminants diets increases MUFA levels while decreasing SFA levels in dairy and meat products and this effect is conducive to consumer health [9,59]. Additionally, Gerasopoulos et al. [34] described decreased n-6/n-3 ratios in the plasma and tissues of piglets fed ensilaged byproducts of olive mill wastewater solids, while other researchers indicated significant increases of unsaturated fatty acids (MUFAs and PUFAs) in the meat of finishing pigs [60,61]. Similarly, Kafantaris et al. [15] reported that the inclusion of grape pomace in piglet diets significantly increased the omega-3 fatty acids and significantly decreased the omega-6/omega-3 ratio compared to control diets (*p* < 0.05). In contrast, another study reported that inclusion of grape pomace solids at a 5% rate in finishing pig diets did not affect the SFA, MUFA, PUFA, n-6 and n-3 PUFA percentages in the meat [62].

## 5. Conclusions

This study evaluated, for the first time, the effects of a novel dietary silage created by combining olive mill, winery and cheese-making by-products of the Greek agro-industry sector on the productive, health and meat quality parameters of weaned pigs. The results indicate that the tested silage containing various bioactive compounds, had no adverse effects on growth performance, zootechnical and health traits of the weaned pigs. In addition, the tested silage did not appear to have adverse effects in gut function and microbial balance. Notable effects were identified on the oxidative stability of the meat and resistance to oxidation was improved. Additionally, the fatty acid analysis of the meat showed that the ratio of n-6/n-3 was improved. Further research is certainly needed to test the bioactivity of these types of silages in pigs in different ratios and age periods to positively influence the environmental impact of pig production in relation to the environmental pollution imposed by the improper disposal of the by-products that were used as the main ingredients of the silage.

## Figures and Tables

**Table 1:** Chemical composition of the examined silage.

Silage Chemical Analysis (As Fed Basis)	
Moisture (%)	42.89
Dry matter (%)	57.11
Ash (%)	1.15
Crude fat (%)	3.21
Crude fiber (%)	2.63
Crude protein (%)	5.51
Total Ca (%)	0.05
Total P (%)	0.18
Mn (mg/kg)	16.95
Fe (mg/kg)	82.48
Cu (mg/kg)	3.21
Zn (mg/kg)	30.43

**Table 2:** Pig diets.

Diets			
**Ingredients (%)**	**Silage—0%**	**Silage—5%**	**Silage—10%**
Maize	43.60	36.97	30.26
Silage	0.00	5.00	10.00
Soybean meal (47% CP)	15.80	16.37	16.94
Barley	20.00	20.00	20.00
Fishmeal 62% CP	3.00	3.00	3.00
Wheat middlings	3.00	3.00	3.00
Soybean oil	2.00	3.06	4.20
Commercial premix 6% *	6.00	6.00	6.00
Whey permeate (4.5% CP)	6.00	6.00	6.00
Zinc oxide	0.30	0.30	0.30
Benzoic acid	0.30	0.30	0.30
**Total**	**100.00**	**100.00**	**100.00**
**Calculated chemical analysis**			
Digestible energy, MJ/kg	13.865	13.862	13.860
Crude protein, %	17.641	17.641	17.640
Dry matter, %	89.029	87.676	86.322
Ash, %	5.450	5.453	5.456
Crude fat, %	4.500	5.434	6.367
Crude fiber, %	2.900	2.860	2.819
ADF, %	3.300	3.259	3.217
NDF, %	9.864	9.613	9.362
Ca, %	0.576	0.577	0.578
Total P, %	0.485	0.480	0.475
Lysine, %	1.177	1.185	1.193
Methionine + Cystine, %	0.740	0.736	0.731

* Provided per kg of diet: 15,000 IU vitamin A, 50 mcg 25-hydroxycholecalciferol, 9.96 mg vitamin E, 10.02 mg vitamin K3, 3 mg vitamin B1, 10.02 mg vitamin B2, 6 mg pantothenic acid, 6 mg vitamin B6, 40.02 mcg vitamin B12, 100 mg vitamin C, 35 mg niacin, 300 mcg biotin, 1.5 mg folic acid, 375 mg choline chloride, 200 mg ferrous sulfate monohydrate, 90 mg copper sulfate pentahydrate, 60 mg manganese sulfate monohydrate, 100 mg zinc sulfate monohydrate, 2 mg calcium iodate, 300 mg sodium selenide, 150 mg L—selenomethionine—Selenium, 1500 FYT 6-phytase, 80 U β-1,4-endoglucanase, 70 U β-1,3 (4)-endoglucanase, 270 U β-1,4-endoxylanase, 5000 mg benzoic acid, 40.8 mg butylated hydroxytoluene, 3.5 mg propyl gallate.

**Table 3:** Effect of silage supplementation on pig performance parameters.

Body Weight on Day (kg)	Diets			SEM	*p*-Value
	Silage 0%	Silage 5%	Silage 10%		
1	8.30	8.32	8.40	0.177	0.975
22	17.46	18.51	18.37	0.404	0.525
40	26.47	26.38	27.64	0.494	0.518
**Weight gain for days (kg)**					
1–22	9.16	10.18	9.97	0.291	0.322
22–40	9.01 ^ab^	7.87 ^a^	9.27 ^b^	0.240	0.038
1–40	18.16	18.06	19.24	0.391	0.401
**Feed intake per pig for days (kg)**					
1–22	14.54	15.97	16.09	NA	NA
22–40	16.86	14.10	15.12	NA	NA
1–40	31.39	30.07	31.21	NA	NA
**FCR for days (kg feed/kg weight gain)**					
1–22	1.587	1.568	1.613	NA	NA
22–40	1.871	1.791	1.632	NA	NA
1–40	1.728	1.665	1.622	NA	NA
**Carcass parameters**					
Carcass weight (kg)	20.33	20.22	20.87	0.732	0.936
Carcass percentage (%)	74.24	74.44	74.52	0.326	0.941

n = 15 pigs per group. NA, Not applicable. ^a,b^ Values with no common superscript differ significantly (*p* ≤ 0.05).

**Table 4:** Effect of silage supplementation on pig intestinal microflora populations.

Ileum Microbes (Log_10_ CFU/g)	Diets			SEM	*p*-Value
	Silage 0%	Silage 5%	Silage 10%		
Total aerobes	5.831	6.467	6.444	0.187	0.304
Total anaerobes	7.138 ^ab^	6.310 ^a^	7.598 ^b^	0.203	0.020
*Enterobacteriaceae*	5.150	4.258	5.347	0.334	0.388
*Enterococcaceae*	0.884	0.000	2.100	0.460	0.164
*Lactobacillaceae*	7.006 ^b^	6.085 ^a^	7.585 ^b^	0.180	<0.001
*Bifidobacteriaceae*	2.985	0.616	1.146	0.431	0.052
**Caecum microbes (Log_10_ CFU/g)**					
Total aerobes	8.893	8.006	7.810	0.322	0.366
Total anaerobes	10.172 ^b^	8.083 ^a^	7.355 ^a^	0.354	<0.001
*Enterobacteriaceae*	5.013	4.167	5.464	0.315	0.245
*Enterococcaceae*	2.333	0.000	1.984	0.520	0.133
*Lactobacillaceae*	10.878 ^c^	9.788 ^b^	8.117 ^a^	0.290	<0.001
*Bifidobacteriaceae*	3.533	1.435	2.271	0.435	0.139

n = 6 pigs per group. ^a,b,c^ Values with no common superscript differ significantly (*p* ≤ 0.05).

**Table 5:** Effect of silage supplementation on pig blood hematological and biochemical parameters.

Hematological Parameters	Diets			SEM	*p*-Value
	Silage 0%	Silage 5%	Silage 10%		
WBC (10^3^/μL)	23.66	19.83	18.52	1.488	0.366
Lymphocytes (%)	32.82	38.63	39.50	2.090	0.388
Monocytes (%)	8.07 ^a^	7.35 ^a^	13.03 ^b^	0.583	0.002
RBC (10^6^/μL)	5.92	5.66	5.48	0.193	0.347
Hct (%)	33.30 ^b^	30.55 ^ab^	28.85 ^a^	0.535	0.013
Hb (g/dL)	12.20 ^b^	11.88 ^ab^	10.93 ^a^	0.175	0.025
**Blood biochemical parameters**					
ALB (g/dL)	2.58	2.52	2.40	0.036	0.146
ALP (μ/L)	327.17	277.83	355.50	13.628	0.094
ALT (U/L)	104.00	78.67	97.50	4.035	0.055
AST (U/L)	67.00	56.83	61.67	3.176	0.445
CHOL (mg/dL)	70.17	77.17	72.00	2.413	0.487
GLU (mg/dL)	97.17 ^b^	80.67 ^ab^	64.00 ^a^	3.037	0.002
TRIG (mg/dL)	39.00	49.67	53.67	3.077	0.197

n = 6 pigs per group. WBC: white blood cells; RBC: red blood cells; HCT: hematocrit; HB: hemoglobin; ALB: albumin; ALP: alkaline phosphatase; ALT: alanine aminotransferase; AST: aspartate aminotransferase; CHOL: cholesterol; GLU: glucose; TRIG: triglycerides. ^a,b^ Values with no common superscript differ significantly (*p* ≤ 0.05).

**Table 6:** Effect of silage supplementation on pig shoulder, ham and belly meat chemical composition and pH.

Shoulder Meat (Triceps Branchii) Chemical Composition (%)	Diets			SEM	*p*-Value
	Silage 0%	Silage 5%	Silage 10%		
Fat	5.12	5.97	4.17	0.372	0.143
Moisture	76.18	75.48	77.14	0.307	0.078
Protein	17.92	18.04	18.06	0.154	0.935
Collagen	1.48	1.50	1.51	0.058	0.985
Ash	1.03 ^ab^	0.97 ^a^	1.14 ^b^	0.2646	0.019
pH	5.57	5.66	5.61	0.051	0.787
**Ham meat (biceps femoris) chemical composition (%)**					
Fat	3.97	3.33	4.16	0.219	0.281
Moisture	76.72	76.86	77.05	0.244	0.873
Protein	18.60	19.38	18.61	0.203	0.198
Collagen	1.19	1.04	1.19	0.032	0.081
Ash	1.02	1.07	1.07	0.028	0.709
pH	5.54	5.48	5.52	0.017	0.384
**Belly meat (external abdominal) chemical composition (%)**					
Fat	9.85	8.74	9.81	0.333	0.325
Moisture	72.18	72.32	71.96	0.328	0.916
Protein	17.35	17.67	17.06	0.114	0.087
Collagen	1.65	1.55	1.48	0.052	0.402
Ash	0.91 ^a^	1.04 ^b^	1.01 ^b^	0.016	0.003
pH	5.52	5.50	5.54	0.014	0.397

n = 6 pigs per group. ^a,b^ Values with no common superscript differ significantly (*p ≤* 0.05).

**Table 7:** Effect of silage supplementation on pig shoulder, ham and belly meat color.

Shoulder Meat Color	Diets			SEM	*p*-Value
	Silage 0%	Silage 5%	Silage 10%		
L*	62.81	61.99	60.28	0.630	0.263
A*	10.60	11.22	12.01	0.301	0.165
B*	9.10	10.13	9.85	0.303	0.386
**Ham meat color**					
L*	64.46	65.49	61.23	1.315	0.416
A*	9.18	9.31	11.42	0.644	0.301
B*	9.85	10.81	9.58	0.381	0.416
**Belly meat color**					
L*	64.39	67.63	66.47	0.802	0.264
A*	11.61	9.27	9.11	0.536	0.094
B*	9.76	10.40	10.36	0.349	0.731

n = 6 pigs per group. L*: lightness; A*: redness; B*: yellowness.

**Table 8:** Effect of silage supplementation on pig shoulder, ham and belly meat microbial populations.

Shoulder Meat Microbes (Log CFU/g)	Diets			SEM	*p*-Value
	Silage 0%	Silage 5%	Silage 10%		
Total mesophilic count	6.426	6.591	6.639	0.0922	0.647
*E. coli*	3.992	4.023	4.227	0.0893	0.540
*S. aureus*	3.213	3.107	3.137	0.1340	0.953
*Staphylococcus* spp.	4.461	4.167	3.958	0.1548	0.443
Sulphite-reducing *Clostridium*	4.235	4.054	3.776	0.1228	0.329
*C. jejuni*	3.830	3.832	3.773	0.0717	0.935
**Ham meat microbes (Log CFU/g)**					
Total mesophilic count	6.677	6.203	5.713	0.1838	0.092
*E. coli*	4.091	4.183	4.279	0.0928	0.741
*S. aureus*	3.204	2.990	3.123	0.1279	0.813
*Staphylococcus* spp.	4.344	3.590	3.802	0.1537	0.113
Sulphite-reducing *Clostridium*	4.172	2.053	2.065	0.3836	0.066
*C. jejuni*	3.857	3.457	3.735	0.0771	0.085
**Belly meat microbes (Log CFU/g)**					
Total mesophilic count	6.710	6.838	6.792	0.0820	0.833
*E. coli*	4.248	3.801	3.954	0.0991	0.178
*S. aureus*	3.384	3.186	3.145	0.1158	0.699
*Staphylococcus* spp.	4.356	4.145	4.113	0.1329	0.750
Sulphite-reducing *Clostridium*	4.382 ^b^	4.352 ^b^	3.690 ^a^	0.1188	0.013
*C. jejuni*	3.828	3.837	4.026	0.0741	0.725

n = 6 pigs per group. ^a,b^ Values with no common superscript differ significantly (*p ≤* 0.05).

**Table 9:** Effect of silage supplementation on pig ham, shoulder and belly meat oxidative stability.

Shoulder Meat	Diets			SEM	*p*-Value
	Silage 0%	Silage 5%	Silage 10%		
Total phenols (g/L)	0.86 ^a^	1.79 ^ab^	2.32 ^b^	0.247	0.022
TBARS (mg MDA/kg)	0.088	0.053	0.047	0.0010	0.390
**Ham meat**					
Total phenols (g/L)	1.75 ^a^	2.24 ^b^	2.36 ^b^	0.091	0.043
TBARS (mg MDA/kg)	0.123 ^b^	0.081 ^ab^	0.068 ^a^	0.0099	0.045
**Belly meat**					
Total phenols (g/L)	1.55 ^a^	2.15 ^b^	2.79 ^c^	0.096	0.001
TBARS (mg MDA/kg)	0.082 ^b^	0.046 ^ab^	0.036 ^a^	0.0074	0.015

n = 6 pigs per group. TBARS: thiobarbituric acid reactive substances; MDA: malondialdehyde. ^a,b,c^ Values with no common superscript differ significantly (*p ≤* 0.05).

**Table 10:** Effect of silage supplementation on pig shoulder meat fatty acid composition.

Shoulder Meat FA (%)	Diets			SEM	*p*-Value
	Silage 0%	Silage 5%	Silage 10%		
(C10:0) Capric	0.10	0.12	0.10	0.009	0.614
(C12:0) Lauric	0.10 ^a^	0.13 ^ab^	0.18 ^b^	0.119	0.011
(C14:0) Myristic	1.41 ^c^	1.07 ^b^	0.76 ^a^	0.065	0.001
(C16:0) Palmitic	22.80 ^c^	18.22 ^b^	15.78 ^a^	0.706	0.001
(C16:1 cis) Palmitoleic	4.10 ^c^	2.74 ^b^	2.13 ^a^	0.200	0.001
(C17:0) Heptadecanoic	0.29 ^b^	0.21 ^a^	0.22 ^a^	0.012	0.006
(C17:1 cis-10) Heptadecenoic cis	0.21 ^b^	0.17 ^b^	0.11 ^a^	0.013	0.002
(C18:0) Stearic	11.07 ^c^	6.08 ^a^	7.20 ^b^	0.519	0.001
(C18:1 cis n9) Oleic	32.66 ^a^	36.68 ^c^	34.52 ^b^	0.399	0.001
(C18:1 n7) Vaccenic	3.24 ^c^	2.67 ^b^	1.94 ^a^	0.129	0.001
(C18:2 n-6c) Linoleic	19.21 ^a^	22.76 ^b^	24.49 ^c^	0.533	0.001
(C20:1 cis n9) cis-11-Eicosenoic	1.52 ^a^	1.68 ^b^	1.78 ^c^	0.027	0.001
(C18:4n-3) Stearidonic	0.64 ^c^	0.53 ^b^	0.46 ^a^	0.020	0.001
(C20:2 cis n-6) cis-11,14-Eicosadienoic	0.65 ^a^	0.67 ^a^	0.79 ^b^	0.017	0.001
C20:3 cis n-6) cis-8,11,14-Eicosatrienoate	0.19 ^a^	0.52 ^b^	0.64 ^c^	0.047	0.001
(C22:5 cis n-3) cis-7,10,13,16,19-Docosapentaenoic acid	0.00 ^a^	0.66 ^b^	0.68 ^b^	0.078	0.001
(C20:3 cis n-3) cis-11-14-17-Eicosatrienoate	0.00 ^a^	0.72 ^b^	0.88 ^c^	0.093	0.001
(C20:4 cis n-6) Arachidonic	0.00 ^a^	0.99 ^b^	1.10 ^c^	0.121	0.001
(C20:5 cis n-3) Cis-5,8,11,14,17-Eicosapentaenoic	0.32 ^a^	0.41 ^b^	0.42 ^b^	0.014	0.001
(C21:5 n-3) Heneicosapentaenoate	0.22 ^a^	0.41 ^b^	0.47 ^c^	0.027	0.001
(C22:6 cis n-3) cis-4,7,10,13,16,19-Docosahexaenoic	0.00 ^a^	2.02 ^b^	1.98 ^b^	0.229	0.001
Σ SFA (Total Saturated FA)	35.77 ^c^	25.83 ^b^	24.23 ^a^	1.238	0.001
Σ MUFA (Total Monounsaturated FA)	41.91 ^b^	44.11 ^c^	40.49 ^a^	0.361	0.001
Σ PUFA (Total Polyunsaturated FA)	21.22 ^a^	29.69 ^b^	34.54 ^b^	1.331	0.001
Σ n-3 (Total omega-3 FA)	1.18 ^a^	4.75 ^b^	4.89 ^c^	0.416	0.001
Σ n-6 (Total omega-6 FA)	20.05 ^a^	24.94 ^b^	27.07 ^c^	0.709	0.001
Ratio n-6/n-3 FA	16.99 ^c^	5.25 ^a^	5.53 ^b^	1.327	0.001

n = 6 pigs per group. FA: Fatty acids; ΣSFA = (C10:0) + (C12:0) + (C14:0) + (C16:0) + (C17:0) + (C18:0); ΣMUFA = (C16:1 cis) + (C17:1 cis-10) + (C18:1 cis n9) + (C18:1 n7) + (C20:1 cis n9); ΣPUFA = (C18:2 n-6c) + (C18:4n-3) + (C20:2 cis n-6) + C20:3 cis n-6) + (C22:5 cis n-3) + (C20:3 cis n-3) + (C20:4 cis n-6) + (C20:5 cis n-3) + (C21:5 n-3) + (C22:6 cis n-3). ^a,b,c^ Values with no common superscript differ significantly (*p ≤* 0.05).

**Table 11:** Effect of silage supplementation on pig belly meat fatty acid composition.

Belly Meat FA (%)	Diets			SEM	*p*-Value
	Silage 0%	Silage 5%	Silage 10%		
(C10:0) Capric	0.07	0.07	0.10	0.009	0.348
(C12:0) Lauric	0.08	0.08	0.09	0.009	0.882
(C14:0) Myristic	1.32	1.30	1.32	0.009	0.614
(C16:0) Palmitic	22.94 ^c^	22.58 ^b^	20.96 ^a^	0.210	0.001
(C16:1 cis) Palmitoleic	2.99 ^b^	2.91 ^a^	3.13 ^c^	0.024	0.001
(C17:0) Heptadecanoic	0.38 ^c^	0.30 ^b^	0.22 ^a^	0.018	0.001
(C17:1 cis-10) Heptadecenoic cis	0.24 ^b^	0.14 ^a^	0.13 ^a^	0.015	0.001
(C18:0) Stearic	12.30 ^c^	11.54 ^b^	11.02 ^a^	0.128	0.001
(C18:1 cis n9) Oleic	34.51 ^c^	27.21 ^a^	31.28 ^b^	0.724	0.001
(C18:1 n7) Vaccenic	2.31 ^a^	2.59 ^b^	2.72 ^c^	0.042	0.001
(C18:2 trans n-6) Linelaidic	0.11 ^b^	0.05 ^a^	0.06 ^ab^	0.010	0.043
(C18:2 n-6c) Linoleic	18.19 ^a^	23.55 ^c^	20.47 ^b^	0.533	0.001
(C18:3 cis n-6) g-Linolenic	0.14 ^a^	0.51 ^c^	0.44 ^b^	0.040	0.001
(C20:1 cis n9) cis-11-Eicosenoic	0.12 ^b^	0.03 ^a^	0.07 ^ab^	0.013	0.002
(C18:3 trans n-3) Linolenic	1.36 ^a^	2.02 ^b^	2.11 ^c^	0.082	0.001
(C18:4 n-3) Stearidonic	0.57	0.55	0.54	0.009	0.434
(C20:2 cis n-6) cis-11,14-Eicosadienoic	0.56 ^a^	0.63 ^b^	0.72 ^c^	0.018	0.001
(C20:3 cis n-6) cis-8,11,14-Eicosatrienoate	0.17 ^a^	0.55 ^b^	0.18 ^a^	0.044	0.001
(C22:5 cis n-3) cis-7,10,13,16,19-Docosapentaenoic acid	0.25 ^a^	0.61 ^b^	0.64 ^b^	0.043	0.001
(C20:3 cis n-3) cis-11-14-17-Eicosatrienoate	0.13 ^a^	0.33 ^b^	0.41 ^c^	0.299	0.001
(C20:4 cis n-6) Arachidonic	0.49 ^a^	0.97 ^b^	0.98 ^b^	0.561	0.001
(C20:5 cis n-3) Cis-5,8,11,14,17-Eicosapentaenoic	0.12 ^a^	0.22 ^b^	0.47 ^c^	0.037	0.001
(C21:5 n-3) Heneicosapentaenoate	0.18 ^a^	0.41 ^b^	0.45 ^b^	0.031	0.001
(C22:6 cis n-3) cis-4,7,10,13,16,19-Docosahexaenoic	0.37 ^a^	0.82 ^b^	1.39 ^c^	0.102	0.001
Σ SFA (Total Saturated FA)	37.18 ^c^	35.94 ^b^	33.77 ^a^	0.342	0.001
Σ MUFA (Total Monounsaturated FA)	40.17 ^c^	32.84 ^a^	37.33 ^b^	0.732	0.001
Σ PUFA (Total Polyunsaturated FA)	22.65 ^a^	31.23 ^c^	28.86 ^b^	0.877	0.001
Σ n-3 (Total omega-3 FA)	2.98 ^a^	4.96 ^b^	6.01 ^c^	0.305	0.001
Σ n-6 (Total omega-6 FA)	19.66 ^a^	26.26 ^c^	22.85 ^b^	0.654	0.001
Ratio n-6/n-3 FA	6.60 ^c^	5.29 ^b^	3.80 ^a^	0.278	0.001

n = 6 pigs per group. FA: Fatty acids; ΣSFA = (C10:0) + (C12:0) + (C14:0) + (C16:0) + (C17:0) + (C18:0); ΣMUFA = (C16:1 cis) + (C17:1 cis-10) + (C18:1 cis n9) + (C18:1 n7) + (C20:1 cis n9); ΣPUFA = (C18:2 n-6c) + (C18:4n-3) + (C20:2 cis n-6) + C20:3 cis n-6) + (C22:5 cis n-3) + (C20:3 cis n-3) + (C20:4 cis n-6) + (C20:5 cis n-3) + (C21:5 n-3) + (C22:6 cis n-3). ^a,b,c^ Values with no common superscript differ significantly (*p ≤* 0.05).

## Data Availability

The data are contained within the article.
